# Performance Degradation Estimation of High-Speed Train Bogie Based on 1D-ConvLSTM Time-Distributed Convolutional Neural Network

**DOI:** 10.1155/2022/5030175

**Published:** 2022-02-26

**Authors:** Junxiao Ren, Weidong Jin, Liang Li, Yunpu Wu, Zhang Sun

**Affiliations:** ^1^School of Electrical Engineering, Southwest Jiaotong University, 999 Xi'an Road, Chengdu 611756, Sichuan, China; ^2^China-ASEAN International Joint Laboratory of Integrated Transportation, Nanning University, 8 Longting Road, Nanning 541699, Guangxi, China

## Abstract

High-speed train bogies are essential for the safety and comfort of train operation. The performance of the bogie usually degrades before it fails, so it is necessary to detect the performance degradation of a high-speed train bogie in advance. In this paper, with two key dampers on the bogie taken as experimental objects (lateral damper and yaw damper), a novel 1D-ConvLSTM time-distributed convolutional neural network (CLTD-CNN) is proposed to estimate the performance degradation of a high-speed train bogie. The proposed CLTD-CNN is an encoder-decoder structure. Specifically, the encoder part of the proposed structure consists of a time-distributed 1D-CNN module and a 1D-ConvLSTM. The decoder part consists of a 1D-ConvLSTM and a simple time-CNN with residual connections. In addition, an auxiliary training part is introduced into the structure to support CLTD-CNN in learning the performance degradation trend characteristic, and a special input format is designed for this structure. The whole structure is end-to-end and does not require expert knowledge or engineering experience. The effectiveness of the proposed CLTD-CNN is tested by the high-speed train CRH380A under different performance states. The experimental results demonstrate the superiority of CLTD-CNN. Compared to other methods, the estimation error of CLTD-CNN is the smallest.

## 1. Introduction

With the rapid development of high-speed trains, the safety of the train operation is widely concerned [1–3]. As a key component, the high-speed train bogie connects the vehicle body and the track and has a huge impact on the safety and comfort of train operation [4]. During the operation, the performance of the bogie inevitably degrades, which may lead to bogie faults [5]. In order to avoid bogie faults caused by performance degradation of the bogie and to ensure the safety of train operation, it is necessary to estimate the performance degradation of the high-speed train bogie.

**Figure 1:**
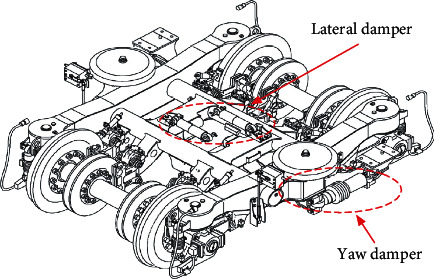
Structure of a high-speed train bogie and bogie key components (lateral damper and yaw damper).

In recent years, research on high-speed train safety has focused more on bogie fault diagnosis [6–11]. However, faults usually occur from the accumulation of performance degradation [12–14]. To get better control of bogie health, the bogie's performance degradation should be detected before faults occur. Therefore, recent studies have focused on bogie performance degradation estimation. The bogie performance degradation results from the performance degradation of bogie key components, the lateral damper and yaw damper [15], as shown in Figure 1. These dampers are able to absorb the shock and vibration caused by uneven wheel-rail contact so as to ensure the safe and comfortable operation of a high-speed train.

Currently, deep learning has achieved great success in many fields because of its strong ability to learn features from big data [16, 17]. Traditional model-based methods are difficult to model high-speed train bogie and train operation [1, 18]. Recent studies about high-speed trains employ deep learning-based methods to process high-speed train vibration signals. Compared with traditional model-based methods, deep learning-based methods are superior [19]. The experimental results of [13, 14] also fully demonstrate that structures based on 1D-CNN or RNN can effectively process high-speed train vibration signals and achieve performance degradation estimation.

However, the studies of [13, 14] still have shortcomings. The usage of samples in the process of model training and testing does not take into account the fact that the performance degradation is decreasing in practice. In other words, the methods proposed in [13, 14] does not take into account the characteristic of the performance degradation trend. Therefore, the characteristic of the performance degradation trend was not effectively utilized in the estimation of damper performance degradation states.

This paper fully considers the above issues and proposes a 1D-ConvLSTM time-distributed CNN (CLTD-CNN), which is an encoder-decoder structure [20] to realize performance degradation estimation of high-speed train bogie, while proposing a new input format for this structure. With this input format, the proposed structure CLTD-CNN is able to learn the characteristics of the performance degradation trend and estimate the unknown postdegradation performance states (the performance states of test samples are not within the range of the performance states of training samples). CLTD-CNN contains three parts: an encoder part, a decoder part, and an auxiliary training part. The encoder part consists of a time-distributed 1D-CNN module [21] and a 1D-ConvLSTM, which encodes the input data in the order of performance states from high to low. The decoder part consists of a 1D-ConvLSTM and a time-CNN [22], which decodes the results obtained by the encoder part and outputs the estimation results. In contrast to long-short-term memory (LSTM) [23], 1D-ConvLSTM is adopted in both the encoder part and the decoder part in order to maintain the temporal and spatial features of input data. The auxiliary training part of CLTD-CNN allows the encoder part to better learn the characteristics of the performance degradation trend. The effectiveness of CLTD-CNN was tested on a high-speed train vibration dataset at 200 km/h. The proposed structure is able to effectively estimate the performance degradation of a high-speed train bogie. In summary, the innovations in this paper are as follows:

1. This paper proposes a novel 1D-ConvLSTM time-distributed CNN (CLTD-CNN) to achieve performance degradation estimation of a high-speed train bogie. This structure can learn the characteristics of the performance degradation trend from early degradation data and estimate unknown performance degradation states.
2. In order to better learn the characteristics of the performance degradation trend, a novel input format is proposed for CLTD-CNN, and an auxiliary training part is introduced to supplement the training of CLTD-CNN.
3. The fully-connected calculation in the LSTM is modified into a one-dimensional (1D) convolutional calculation, turning the LSTM into 1D-ConvLSTM. The 1D-ConvLSTM learns the characteristics of the performance degradation trend without destroying the spatial features of the samples during the training.

The outline of this paper is organized as follows.  Section 2 presents the recent works.  Section 3 presents the proposed 1D-ConvLSTM time-distributed CNN in detail. Experiments are demonstrated in Section 4, with a brief introduction of the adopted experimental data.  Section 5 concludes this paper.

## 2. Literature Review

### 2.1. High-Speed Train Bogie Fault Diagnosis and Performance Degradation Estimation

In the field of high-speed train bogie fault diagnosis and performance degradation estimation, a number of review studies [1, 2, 19] summarize the results of recent years and future directions. These studies compared model-based methods with deep learning methods and pointed out that deep learning methods based on big data are currently more advantageous.

Model-based methods made some contributions, focusing more on the assessment of the bogie as a whole. Lu et al. [9] proposed an accelerated life test (ALT) method to predict the fatigue life of a full-scale bogie frame by performing fatigue tests on a real bogie platform. Ji et al. [11] collected bogie fatigue key points (FKPs) data for calculating the actual damage spectrum and load spectrum damage and evaluated the fatigue damage of high-speed train bogies based on the damage consistency load spectra (DCLS) calibration method. Compared with model-based methods, the research on high-speed train bogie fault diagnosis based on deep learning methods can identify the status of a component on the bogie more accurately. Hu et al. [6] adopted deep neural networks to adaptively extract fault information from the signal spectrum to achieve detection of high-speed train bogies and obtain very high diagnostic accuracy. Su et al. [7] proposed a residual-squeeze net based on convolutional neural networks to achieve fault classification of high-speed train bogies. Wu et al. [8] proposed a multiview fault diagnosis architecture based on variable mode decomposition and an enhanced multiscale convolutional neural network to achieve bogie fault diagnosis, taking into full consideration the complexity of the vibration signal components of high-speed trains. Chen et al. [24] proposed a CapsNet-based model to achieve the identification and classification of seven operating conditions of a high-speed train bogie, consisting of single and compound faults. Different deep learning methods to achieve high-speed train bogie fault diagnosis are explained in [4, 5, 10, 25, 26]..

High-speed train bogie performance degradation can be seen as an early fault. Research on performance degradation estimation is in its infancy, and relatively few studies have been carried out. Based on the time-frequency analysis, Ren et al. [13] found that the high-speed train vibration signal contains different frequency components and proposed a multiscale depth separable convolutional neural network (SDS-CNN) to realize the performance degradation estimation of lateral dampers. Qin et al. [14] considered the intrinsic link between bogie fault type and performance degradation and proposed a novel multiple convolutional recurrent neural network (M-CRNN) for simultaneous diagnosis of fault types and performance degradation states. These studies demonstrate the effectiveness of deep learning methods for performance degradation estimation, but they have some shortcomings that need improvement.

The performance degradation samples employed in the training process of [13, 14] covered the performance states of 100%–40%, and the performance states of the samples employed for testing also ranged from 100% to 40%. However, the performance degradation of lateral damper and yaw damper is decreasing during the service of the high-speed train, so there is no way to obtain vibration signal samples with serious performance degradation states in a short period of time from currently operating trains. That is, the samples utilized in the model training process should be the samples with early performance degradation states, such as 100%, 90%, 80%, and 70%. Moreover, in practical situations, it is not possible to collect samples with serious performance degradation states (e.g., 60%, 50%, 40%, etc.) for model training because of safety reasons. In addition, after the model training is completed, the performance of the dampers will continue to degrade during continuous operation, so the performance states of the test samples should not be within the range of the performance states of the training samples. For example, the performance states of the test samples are the performance states after further degradation based on the performance states of the training samples.

Besides the above shortcoming, there is another one in [13, 14]. The model training in [13, 14] fully considers that the performance degradation of the bogie damper components is slow and that the slow degradation means a single vibration signal sample (sampling frequency of 243 Hz and sampling duration of 1 s) cannot show the performance degradation trend. Therefore, a single sample is considered to directly correspond to one performance state. The study [14] analyzed the samples with different performance states and demonstrated that, between samples with different performance degradation states, there exists the characteristic of degradation trend.

In general, Table 1 provides a comprehensive summary of these recent works on high-speed train bogie fault diagnosis and performance degradation estimation.

### 2.2. Deep Learning

In recent years, the great potential of deep learning was first demonstrated in the fields of image and video [16, 17, 20, 27, 28]. Later on, with the development of 1D-CNN and RNN, deep learning has received wide attention and achieved great success in the field of temporal signal processing [22, 23]. Qiao et al. [21] proposed an end-to-end hybrid deep learning framework for machine health monitoring based on multisensor time-series data. Meng and Zhu [29] proposed a convolution-based long-short-term memory (CLSTM) network to process mining site vibration data to predict the remaining useful life of rotating machinery. Xiang et al. [30] employed an isometric mapping algorithm to construct health indicators based on the statistical properties of gears. With this basis, a long-short-term memory neural network with attention-guided ordered neurons (LSTM-AON) was proposed to achieve an accurate prediction of the remaining useful life (RUL) of gears. The wide application and great success of deep learning in the field of signal processing are demonstrated in [31–33].

In general, these recent works on deep learning are summarized in Table 2.

## 3. Proposed 1D-ConvLSTM Time-Distributed CNN

**Figure 2:**
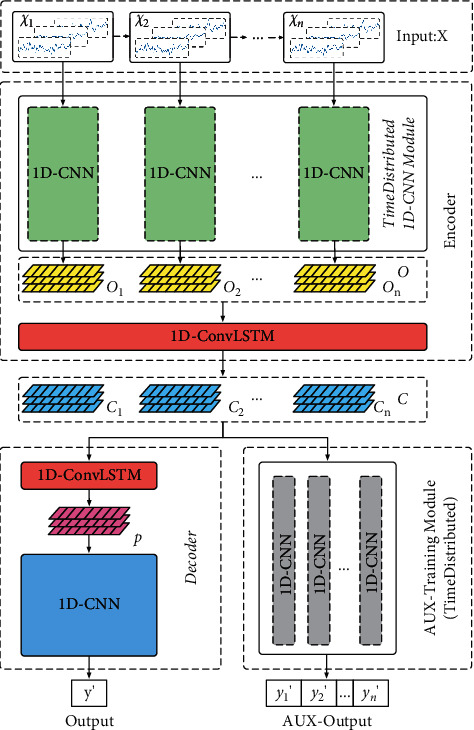
Overall structure of proposed CLTD-CNN.

In order to make performance degradation estimation of high-speed train bogie more in line with real practice, CLTD-CNN (an encoder-decoder structure) is proposed in this paper, as shown in Figure 2. Furthermore, a novel data input format is proposed for this structure. The input data is composed of *n* samples arranged in decreasing order of performance states. The proposed CLTD-CNN contains three parts: an encoder part, a decoder part, and an auxiliary training part. The encoder part contains a time-distributed 1D-CNN module and a 1D-ConvLSTM. The decoder part contains a 1D-ConvLSTM and a simple time-CNN with residual connections [27]. Meanwhile, CLTD-CNN introduces an auxiliary training part with a time-distributed property to supplement the training, allowing the encoder part to better learn the characteristics of the performance degradation trend. The proposed structure estimates the current performance degradation state by combining the information from the samples of early performance degradation states. The estimation result *y*′ can be expressed as follows:

(1) $$\begin{array}{c} y^{\prime }=\underset{y}{\arg\max}\;p\left(y|x_{1},x_{2},\ldots ,x_{i},\ldots ,x_{n}\right), \end{array}$$

where *x*_*i*_ represents performance degradation sample, *y* represents the actual performance state of the *n* th sample, and *y*′ represents the estimation result of the *n* th sample (*x*_*n*_).

It is worth noting that because the high-speed train vibration data adopted in this paper are the same as those in [13, 14], the hyperparameters (such as the size and number of convolutional kernels, the length of the convolutional stride, and the number of nodes in the fully-connected layer) of the proposed CLTD-CNN in Figure 2 have been chosen with reference to the structures (SDS-CNN and M-CRNN) in [13, 14]. The details of these hyperparameters are presented accordingly in this section. As the structure of the input *X* is one of the innovations in this paper, the step length *n* of the input *X* is one of the most important hyperparameters in the paper. The size of *n* is obtained experimentally, as seen specifically in the experimental Section 4.2.

### 3.1. Novel Data Input Format

A novel data input format is proposed in this paper. This format enables CLTD-CNN to make effective use of early degradation data to learn the characteristics of the performance degradation trend. In [13, 14], performance degradation samples are taken as separate inputs. The data input formats of [13, 14] ignore the characteristics of the performance degradation trend, which makes the methods proposed in [13, 14] unable to accurately estimate the unknown further degraded performance states.

**Figure 3:**
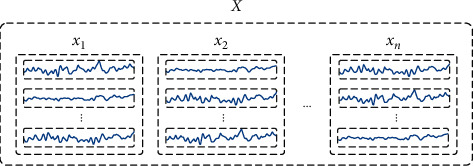
The format of the input *X*.

In the data input format proposed in this paper, an individual vibration signal sample is denoted as *x*_*i*_. The size of the sample *x*_*i*_ is *l* × *c*, where *l* is the sample length and *c* is the number of signal channels. *n* samples in decreasing order of performance states are selected to form a novel input *X*=[*x*_1_, *x*_2_,…*x*_*i*_,…, *x*_*n*_], as shown in Figure 3. Each *X* matches two labels, *y*=*y*_*n*_ and *Y*=[*y*_1_, *y*_2_,…*y*_*i*_,…, *y*_*n*_], where *y* corresponds to *x*_*n*_ (the performance degradation state that needs to be estimated). *y*_*i*_ in *Y* corresponds to the performance degradation state of each *x*_*i*_ in *X* (*Y* is employed to calculate the *AuxLoss* in the auxiliary training part, as seen in 3.3). In the estimation of the further degraded performance states, the performance states from *x*_1_ to *x*_*n*−1_ are known, and they represent decreasing performance states. *x*_1_ is the sample with the highest performance state. *x*_*n*_ is the sample with the lowest performance state that needs to be estimated. This means that *x*_*i*_ corresponds to a true label satisfying *y*_1_ > *y*_2_ > …>*y*_*i*_ > …>*y*_*n*_. *n* represents step length of input *X*. Such an input format allows historical data of these early degraded samples (*x*_1_ to *x*_*n*−1_) to be utilized in the estimation of *x*_*n*_.

The performance degradation order from *x*_1_ to *x*_*n*_ is regarded as a process of performance degradation in temporal order, so input *X* can be regarded as containing temporal features of performance degradation. Sample *x*_*i*_, a component in input *X*, is essentially a multichannel vibration signal. This vibration signal also contains the spatial features when processed by 1D-CNN. In comparison with video data [28], input *X* proposed in this paper can be regarded as the data in a spatiotemporal sequence format, containing features in both temporal and spatial dimensions. Such a data format allows the proposed structure to not only learn the features of *x*_*n*_ itself, but also make full use of the historical data of these early degraded samples from *x*_1_ to *x*_*n*−1_ to achieve the characteristic learning of the performance degradation trend.

### 3.2. Encoder Part

**Figure 4:**
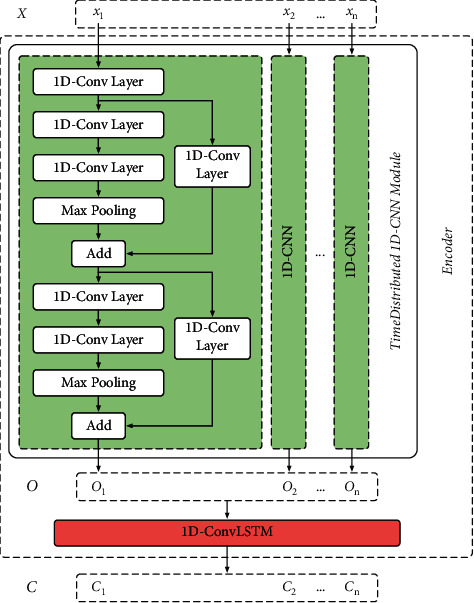
Detailed structure of encoder part.

The encoder part is a seq2seq structure [31] consisting of a time-distributed 1D-CNN module and a 1D-ConvLSTM. This seq2seq structure can simultaneously process each individual *x*_*i*_ in input *X* and can learn the characteristics of the performance degradation trend from *x*_1_ to *x*_*n*_, as shown in Figure 4. The seq2seq structure is usually adopted in the field of machine translation [34]. It normally consists of one or more RNNs (LSTM and GRU). The input data of the machine translation domain is a vector of word representations. In contrast, each sample *x*_*i*_ utilized in this paper is a multichannel vibrational signal with high data dimensionality and redundancy. If *X* is input directly into the 1D-ConvLSTM, the 1D-ConvLSTM cannot effectively encode each *x*_*i*_ in input *X*, resulting in a large estimation error. Therefore, a time-distributed 1D-CNN module is adopted in this part to extract the features from the input *X* first. The output *O* of this module is then fed into 1D-ConvLSTM to achieve effective encoding.

#### 3.2.1. Details of Time-Distributed 1D-CNN Module in Encoder Part

Input *X*=[*x*_1_, *x*_2_,…*x*_*i*_,…, *x*_*n*_] adopted in this paper can be regarded as a kind of spatio-temporal sequence data with size *n* × *l* × *c*, where the size of the sample *x*_*i*_ is *l* × *c*. Regular 1D-CNN cannot directly extract the features of input *X* which contains 3 dimensions. Therefore, this paper adopts a time-distributed 1D-CNN module for feature extraction of input *X*, as shown in Figure 4. This module is able to extract features separately from each *x*_*i*_ in input *X* without destroying the sequence format of the input *X* so that the output *O* still contains a spatio-temporal sequence format. The output *O* can be utilized directly as input of the 1D-ConvLSTM in the encoder part. The time-distributed 1D-CNN module gives traditional CNN models a sequence-to-sequence capability, increasing the dimensionality of the model. Therefore, this module offers more possibilities to deal with complex data structures. The hyperparameters of 1D-CNN in Figure 4 are shown in Table 3.

#### 3.2.2. Details of 1D-ConvLSTM in Encoder Part

**Figure 5:**
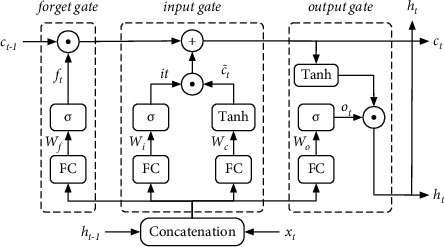
Detailed structure of LSTM.

Currently, traditional LSTM [35] is widely applied in sequence-related problems. Compared with RNN, LSTM incorporates an oblivious mechanism, which avoids the problem of RNN gradient explosion to a certain extent [36]. The illustration of the inner structure of LSTM is shown in Figure 5, in which the calculation formulas are as follows:

(2) $$\begin{array}{c} \begin{array}{c} f_{t}=\sigma \left(W_{f}\cdot \left[h_{t-1},x_{t}\right]+b_{f}\right)\\ i_{t}=\sigma \left(W_{i}\cdot \left[h_{t-1},x_{t}\right]+b_{i}\right)\\ {\tilde{c}}_{t}=\text{tanh}\left(W_{c}\cdot \left[h_{t-1},x_{t}\right]+b_{c}\right)\\ c_{t}=f_{t}⊙c_{t-1}+i_{t}⊙{\tilde{c}}_{t}\\ o_{t}=\sigma \left(W_{o}\cdot \left[h_{t-1},x_{t}\right]+b_{o}\right)\\ h_{t}=o_{t}⊙\text{tanh}\left(c_{t}\right) \end{array}, \end{array}$$

where · represents fully-connected calculation, ⊙ represents Hadamard product, and *σ* represents activation function Relu.

As can be seen from (2), the internal computation of LSTM is implemented by employing a fully-connected calculation. Although LSTM has been proven to be effective in dealing with time series, it is not effective in dealing with spatio-temporal sequences containing spatial features. The main disadvantage of LSTM in processing spatio-temporal sequences is that LSTM employs fully-connected calculation in the transitions both from input to state and from state to state, resulting in spatial features not being encoded [29]. If LSTM is adopted directly to process input *X*, the spatial features of each *x*_*i*_ in input *X* will be ignored.

**Figure 6:**
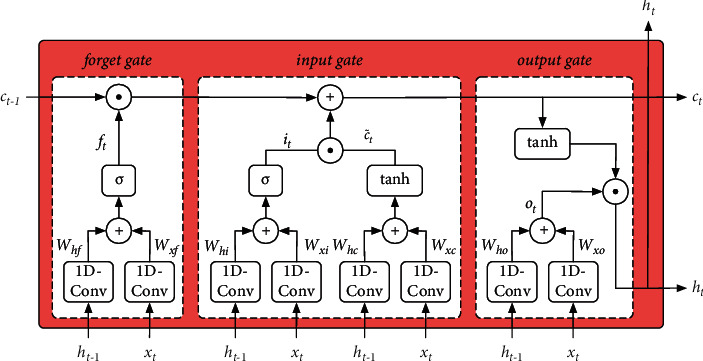
Detailed structure of 1D-ConvLSTM.

1D-CNN has been proved in various studies to be effective in processing high-speed train vibration signals [4–8, 13, 14, 24]. That means 1D-CNN can effectively learn the spatial features of the sample *x*_*i*_ in input *X*. Therefore, in this paper, we consider replacing the fully-connected calculation utilized in the transition both from input to state and from state to state in LSTM with a 1D convolutional calculation so that the improved LSTM can better process sample *x*_*i*_ and encode the spatial features contained in sample *x*_*i*_. The improved LSTM is referred to as 1D-ConvLSTM, and its intrinsic structure is shown in Figure 6, in which the calculation formulas are as follows:

(3) $$\begin{array}{c} \begin{array}{c} f_{t}=\sigma \left(W_{xf}*x_{t}+W_{hf}*h_{t-1}\right)\\ i_{t}=\sigma \left(W_{xi}*x_{t}+W_{hi}*h_{t-1}\right)\\ {\tilde{c}}_{t}=\text{tanh}\left(W_{xc}*x_{t}+W_{hc}*h_{t-1}\right)\\ c_{t}=f_{t}⊙c_{t-1}+i_{t}⊙{\tilde{c}}_{t}\\ o_{t}=\sigma \left(W_{xo}*x_{t}+W_{ho}*h_{t-1}\right)\\ h_{t}=o_{t}⊙\text{tanh}\left(c_{t}\right) \end{array}, \end{array}$$

where *∗* represents 1D convolutional calculation, ⊙ represents Hadamard product, and *σ* represents activation function Relu. *x*_*t*_ and *h*_*t*−1_ are first computed separately during the state transition of 1D-ConvLSTM by one-dimensional convolution, and then summed. In contrast to (2), *x*_*t*_ and *h*_*t*−1_ are first concatenated and then computed via fully-connected calculation in LSTM. The hyperparameters of 1D-ConvLSTM in the encoder part are shown in Table 4.

### 3.3. Auxiliary Training Part

**Figure 7:**
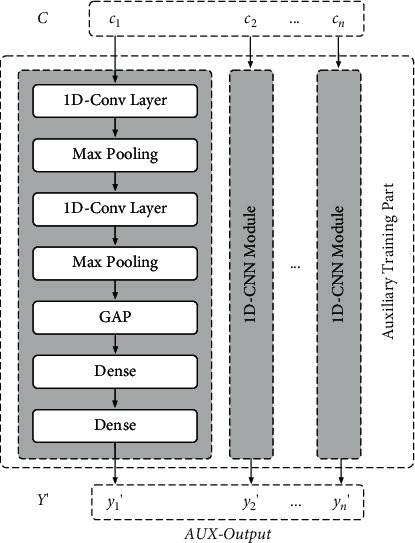
Detailed structure of auxiliary training part.

In this paper, the auxiliary training part is introduced to assist the encoder part in learning the characteristics of the performance degradation trend between samples and is a simplified time-CNN [22] with time-distributed property [21], as shown in Figure 7. The input of this part is feature code *C*=[*c*_1_, *c*_2_,…*c*_*i*_,…, *c*_*n*_] obtained by the encoder part. The output of this part is a sequence *Y*′=[*y*_1_′, *y*_2_′,…*y*_*i*_′,…, *y*_*n*_′], denoted as *AuxResult*, where *y*_*i*_′ is the auxiliary estimation result, corresponding to sample *x*_*i*_. By reducing the error between *Y*′ and the true performance state *Y*=[*y*_1_, *y*_2_,…*y*_*i*_,…, *y*_*n*_] during training, the encoder part is able to learn more accurately the features of different performance state samples and the characteristics of the performance degradation trend. The hyperparameters of 1D-CNN in auxiliary training part are shown in Table 5.

The auxiliary training part makes the output sequence *Y*′ closer to the true performance state *Y* during the training process. This is a regression problem. Therefore, the mean square error (MSE) is adopted as the loss function for this part, denoted as *AuxLoss* which is determined as follows:

(4) $$\begin{array}{c} \text{AuxLoss}=\text{MSE}\left(Y^{\prime },Y\right)=\frac{1}{m}\cdot \frac{1}{n}\sum_{j=1}^{m}\sum_{i=1}^{n}{\left(y_{i}^{\prime }-y_{i}\right)}^{2}, \end{array}$$

where *m* represents the number of input *X* and *n* represents the step length of *Y*(*Y*′).

### 3.4. Decoder Part

**Figure 8:**
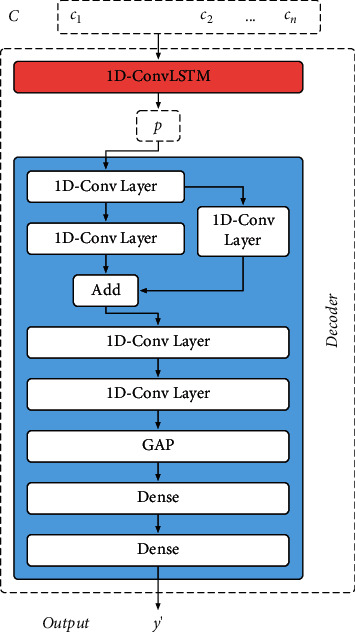
Detailed structure of decoder part.

The decoder part contains a 1D-ConvLSTM (whose structure is the same as that of the 1D-ConvLSTM in the encoder part) and a simple time-CNN with residual connections [27]. This part decodes feature code *C* and outputs performance degradation estimation result *y*′. The detailed structure of the decoder part is shown in Figure 8. In contrast to the encoder part, the 1D-ConvLSTM in the decoder part does not output a sequence, but a feature map *p* which contains not only features of sample *x*_*n*_ itself (the sample whose performance state needs to be estimated) but also the performance degradation trend features from *x*_1_ to *x*_*n*−1_. Then, *p* is decoded by a 1D-CNN structure with residual connections to obtain the estimation result *y*′. Combined with equation (1), *y*′ can be defined as follows:

(5) $$\begin{array}{c} \begin{array}{cc} y^{\prime } & =\underset{y}{\text{arg}\max}\;p\left(y|x_{1},x_{2},\ldots ,x_{n}\right)\\ \approx \underset{y}{\text{arg}\max}\;p\left(y|f_{\text{encoding }}\left(x_{1},x_{2},\ldots ,x_{n}\right)\right)\\ \approx g_{\text{decoding }}\left(f_{\text{encoding }}\left(x_{1},x_{2},\ldots ,x_{n}\right)\right) \end{array}, \end{array}$$

where *f*_encoding_ represents the calculation of the encoder part and *g*_decoding_ represents the calculation of the decoder part. The hyperparameters of the decoder part are shown in Table 6.

The output of the decoder part is a specific value *y*′. This is still a regression problem. Therefore, this part also employs *MSE* as the loss function, denoted as ResultLoss. The detail of ResultLoss is as expressed as follows:

(6) $$\begin{array}{c} \text{ResultLoss}=\text{MSE}\left(y^{\prime },y\right)=\frac{1}{m}\sum_{i=1}^{m}{\left(y_{i}^{\prime }-y_{i}\right)}^{2}, \end{array}$$

where *y* represents the true label and *m* represents the number of input X. For the proposed CLTD-CNN, the overall loss function, denoted as TotalLoss, contains two components, ResultLoss and AuxLoss. The definition of TotalLoss is as expressed as follows:

(7) $$\begin{array}{c} \text{TotalLoss}=\left(1-\lambda \right)\cdot \text{ResultLoss}+\lambda \cdot \text{AuxLoss}, \end{array}$$

where *λ* represents weighting coefficients. According to the experimental results in 3.3, *λ* is taken as 0.2 in this paper.

## 4. Experiment

In this section, the proposed structure is well investigated by focusing on two key bogie components, the lateral damper and the yaw damper, and the effectiveness and superiority of the proposed structure are demonstrated and proved through experiments. The experimental results show that the proposed structure can be utilized to estimate unknown further degraded performance states by adopting historical data of early degradation. The experimental data adopted in the experiments come from high-speed train vibration signal datasets [14]. The mean absolute error (MAE) and root mean square error (RMSE) have been employed as metrics to evaluate the performance of the structures. MAE better reflects the actual states of errors in the estimated values, which is as given as follows:

(8) $$\begin{array}{c} \text{MAE}\left(y^{\prime },y\right)=\frac{1}{m}\sum_{i=1}^{m}\left|y_{i}^{\prime }-y_{i}\right|, \end{array}$$

where *y*_*i*_′ represents the estimation result, *y*_*i*_ represents the true label, and *m* represents the number of input *X*. RMSE is more sensitive to values with larger errors and is as expressed as follows:

(9) $$\begin{array}{c} \text{RMSE}\left(y^{\prime },y\right)=\sqrt{\frac{1}{m}\sum_{i=1}^{m}{\left(y_{i}^{\prime }-y_{i}\right)}^{2}}, \end{array}$$

All experiments were performed in Python (applying Keras, TensorFlow) on a PC with 2.80 GHz × 4CPU, 32 GB RAM, and NVIDIA 1080Ti GPU.

### 4.1. Data Description

**Figure 9:**
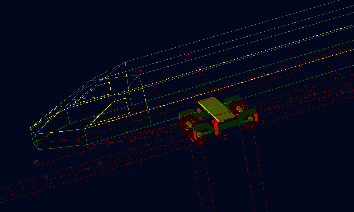
Simulation model of CRH380A.

**Figure 10:**
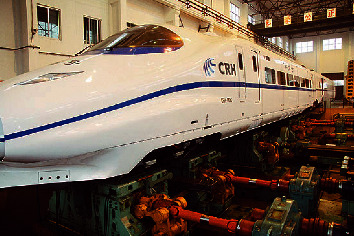
Actual rolling and vibration test rig of the vehicle in the key laboratory of rail transportation of Southwest Jiaotong University.

**Figure 11:**
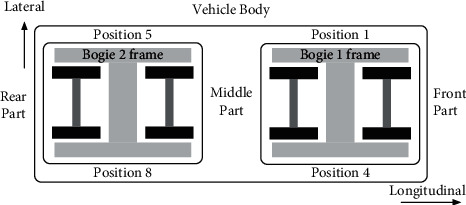
Location of sensors.

**Figure 12:**
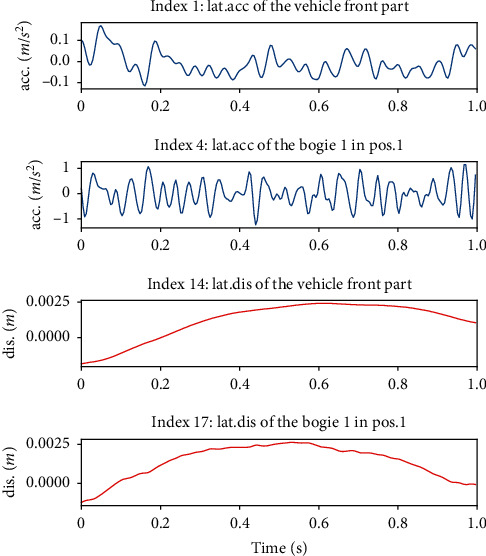
Portions of an acceleration signal sample and portions of a displacement signal sample (there are a total of 58 channels in the acceleration signal sample and displacement signal samples, respectively. Here, randomly demonstrated two channels of each sample are given.).

For safety reasons, the high-speed train vibration data adopted in this paper was obtained through simulation by applying Simpack [7, 25, 26] and came from the same simulation platform as the data adopted in [13, 14]. The high-speed train model employed for simulation is CRH380A, and the actual measured track spectrum of the Wuhan-Guangzhou line is employed as the simulation track. The CRH380A model is provided by the Key Laboratory of Rail Transportation of Southwest Jiaotong University and is shown in Figure 9. The relevant parameters of the CRH380A model are set based on the actual rolling and vibration test rig of the vehicle in the key laboratory of rail transportation of Southwest Jiaotong University, as shown in Figure 10. The sensor settings for the vibration signals of the high-speed train in the simulation model are also the same as those of the actual rolling and vibration test rig of the vehicle. There are 58 sensors in total, 29 acceleration sensors, and 29 displacement sensors. The sampling frequency of each sensor is 243 Hz. The details of all the sensors are shown in Table 7. The location of the sensors is shown in Figure 11. The vibration signal samples utilized in this paper are sampled at 1 s, and the size of these samples is 243 × 58. Portions of an acceleration signal sample and portions of a displacement signal sample are demonstrated in Figure 12.

In the experiments, the training and test sets are set up to fully simulate high-speed train vibration signals collected in real-world situations (the performance degradation state is decreasing in the actual signal collection process, and the state that needs to be estimated is the one that is further degraded from the previous degradation. For example, the performance state of the test set is smaller than the performance state of the training set and is not in the range of the training set's performance states.). Combined with the input data format proposed in this paper, the details of the lateral damper training set and the corresponding test set are shown in Table 8. The details of the yaw damper training set and the corresponding test set are shown in Table 9. It is worth noting that 20% of the training set is randomly reserved as a validation set in the training process.

A particular input *X*=[*x*_1_, *x*_2_,…*x*_*i*_,…, *x*_*n*_] in training set contains *n* individual samples, *n*=4 in this paper (as described in 3.3). The input *X*′=[*x*_1_′, *x*_2_′,…*x*_*i*_′,…, *x*_*n*_′] in test set has the same format as *X*, where the performance state of sample *x*_*n*_′ needs to be estimated and the performance state corresponding to *x*_*n*_′ is not in the range of training set performance states. For example, as shown in Table 8, an input *X* of lateral damper in training set contains 95%, 90%, 85%, and 80% (4 performance states in total). The corresponding input *X*′ of lateral damper in test set contains 90%, 85%, 80%, and 75% (4 performance states in total). Among these, 90%, 85%, and 80% belong to the early degraded historical data that appear in the training set. On estimating the performance state of 75% in input *X*′, the early degraded historical data (90%, 85%, and 80%) was adopted.

### 4.2. Experiments on Step Length *n* of Input *X*

**Figure 13:**
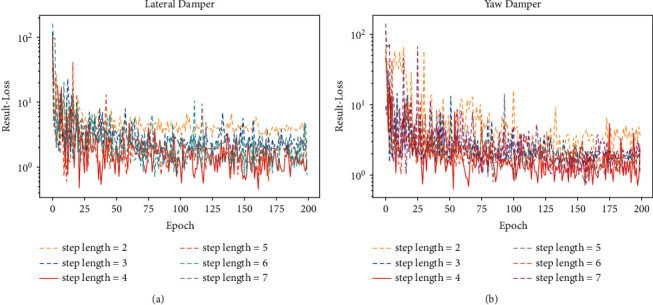
Validation loss of experiments on step length *n*. (a) Validation loss of lateral damper. (b) Validation loss of yaw damper.

In this section, we investigate the effect that step length *n* has on the estimation error. In the proposed structure, both encoder part and decoder part contain 1D-ConvLSTM which is an improved version of LSTM. Although LSTM was proposed to alleviate the problem of gradient descent as well as gradient explosion in RNN [23], it does not mean that LSTM can really handle sequences with a long distance. Therefore, step length *n* should not be too long for sequences with a long distance will still cause gradient explosion. However, if step length *n* is too short, it will not be possible to make full use of the historical data from early degradation during model training to learn the characteristic of performance degradation trend. Experiments in this section were conducted by employing different step length *n* to observe the effect on the estimation results. The results of validation loss during training are shown in Figure 13, and the estimation results are shown in Table 10. The experimental results demonstrate that the estimation results are not satisfactory when step length *n* is too long, and that the estimation result error is relatively large when step length *n* is too short. Therefore, considering the estimation results of lateral damper and yaw damper, step length *n* in this paper is selected as 4.

### 4.3. Ablation Experiments of CLTD-CNN

**Figure 14:**
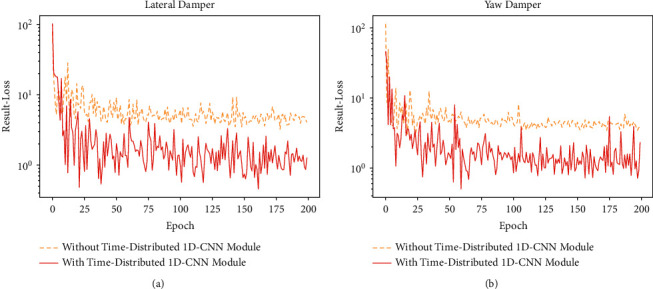
Validation loss of experiments on time-distributed 1D-CNN module. (a) Validation loss of lateral damper. (b) Validation loss of yaw damper.

This section first investigates the rationality of applying time-distributed 1D-CNN module before 1D-ConvLSTM in the encoder part. According to Section 3.2.2, in the transition both from input to state and from state to state, 1D-ConvLSTM only adopts one 1D-CNN layer for feature extraction. If 1D-ConvLSTM is directly applied to process input *X* without applying time-distributed 1D-CNN module to extract the features of input *X* in advance, the features of input *X* may not be extracted effectively, resulting in large estimation error. Therefore, we compare the estimation error with or without applying time-distributed 1D-CNN module before 1D-ConvLSTM. The results of validation loss during training are shown in Figure 14, and the estimation results are shown in Table 11. The experimental results show that without applying time-distributed 1D-CNN module in the encoder part, 1D-ConvLSTM cannot effectively extract the features of input *X*, resulting in large estimation error, and that when time-distributed 1D-CNN module is applied before 1D-ConvLSTM to extract the features of input *X*, the estimation error is significantly reduced.

**Figure 15:**
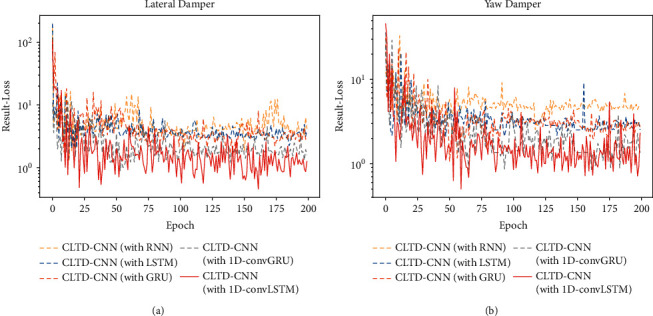
Validation loss of experiments on different RNN structures. (a) Validation loss of lateral damper. (b) Validation loss of yaw damper.

This section then investigates the advantages of applying 1D-ConvLSTM in the proposed CLTD-CNN. Other RNN structures (RNN [32], LSTM, GRU [37], and 1D-ConvGRU [38]) are employed instead of 1D-ConvLSTM in both encoder part and decoder part. The results of validation loss during training are shown in Figure 15, and the estimation results are shown in Table 12. In the experiments, when employing RNN, LSTM, and GRU, the output of time-distributed 1D-CNN module is transformed to fit the input formats of RNN, LSTM, and GRU. As shown in Table 12, when processing data with a spatio-temporal sequence format such as input *X*, the estimation results of RNN, LSTM, and GRU are significantly worse than these of 1D-ConvLSTM and 1D-ConvGRU (1D-ConvGRU is modified from GRU. The fully-connected calculation inside GRU is replaced by 1D convolutional calculation.). Compared with 1D-ConvGRU, the estimation result error of 1D-ConvLSTM is smaller.

**Figure 16:**
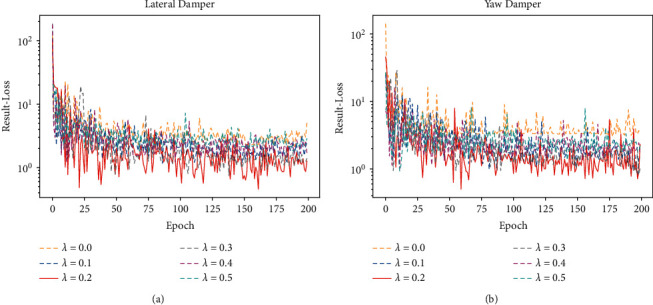
Validation loss of experiments on *λ*. (a) Validation loss of lateral damper. (b) Validation loss of yaw damper.

Finally, this section investigates the effect of the auxiliary training part on the estimation results and the most suitable value of weight *λ*. As can be seen in (7), weight *λ* controls the size of *AuxLoss* in the auxiliary training part. Therefore, adjusting the size of weight *λ* can reflect the effect of auxiliary training part on the estimation results. The results of validation loss during training are shown in Figure 16, and the estimation results are shown in Table 13. It can be seen from the figure that, without the auxiliary training part (weight *λ*=0), the error of estimation result is large. As weight *λ* increases, the error gradually becomes smaller. When weight *λ* is too large, the error becomes larger again. The experimental results demonstrate that the auxiliary training part does have a positive effect on the training of the encoder part. However, the choice of weight *λ* needs to be determined experimentally. Weight *λ* should not be too large or too small. Combined with the experimental results, weight *λ* in this paper is taken as 0.2.

### 4.4. Comparison Experiments

**Figure 17:**
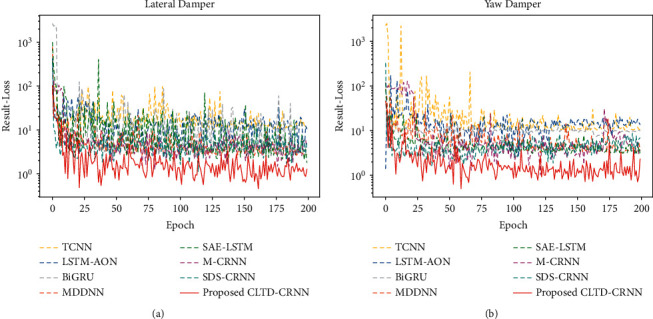
Validation loss of comparison experiments: (a) validation loss of lateral damper, and (b) validation loss of yaw damper.

This section first presents comparison experiments between the proposed CLTD-CNN and the recent state-of-the-art methods M-CRNN [14] and SDS-CNN [13] in the same field. It is worth noting that, currently, studies on the bogie performance degradation of high-speed trains are starting to receive attention and are relatively scarce. Therefore, in this section, the state-of-the-art methods from other fields are also introduced for comparisons, such as methods TCNN [39], LSTM-AON [30], BiGRU [33], MDDNN [40], and SAE-LSTM [41] on bearing performance degradation estimation. The comparison results are shown in Figure 17 and Table 14. The superiority of the CLTD-CNN proposed in this paper is proved by the experimental results.

Methods from other fields, such as TCNN, LSTM-AON, BiGRU, MDDNN, and SAE-LSTM, have some reference value. But their structures are not suitable for dealing with high-speed train vibration signals. The CLTD-CNN proposed in this paper fully considers the characteristics of high-speed train vibration signals and has obvious advantages in estimation errors compared with these methods. Compared with M-CRNN and SDS-CNN of the same field for bogie performance degradation of high-speed trains, the proposed CLTD-CNN still has significant advantages. This is because M-CRNN and SDS-CNN are built by utilizing the same range of performance states included in both the training and test sets. When these two methods are experimented with unknown performance states that do not belong to the range of training set performance states, they are unable to accurately estimate the unknown performance states (for example, the samples of the training set cover the performance states between 100% and 80%, while the samples utilized for testing are with performance states less than 80%). The CLTD-CNN proposed in this paper takes the above issue into account, and the experimental results demonstrate that CLTD-CNN can effectively adopt the historical data of early degradation to accurately estimate the unknown performance states.

Moreover, in Table 14, a comparison of the time complexity of these methods is also provided by presenting floating-point operations (FLOPs), average training time per epoch, and inference time. Compared to relatively simple structures like TCNN and BiGRU, the proposed CLTD-CNN does not have an advantage in time complexity, but the estimation error of the proposed CLTD-CNN is much smaller than that of TCNN and BiGRU. The time complexity of the proposed CLTD-CNN is acceptable when compared to structures with similar or higher time complexity, such as LSTM-AON, MDDNN, SAE-LSTM, M-CRNN, and SDS-CNN. In addition, CLTD-CNN fully considers the characteristics of the high-speed train vibration signal. Therefore, the proposed CLTD-CNN has a significant advantage in estimation error, with acceptable time complexity.

## 5. Conclusion

This paper proposes a novel 1D-ConvLSTM time-distributed convolutional neural network (CLTD-CNN) to realize the performance degradation estimation of a high-speed train bogie by experimenting on two key bogie components (lateral damper and yaw damper). At the same time, this paper proposes a novel input format for CLTD-CNN. With this input format, CLTD-CNN is able to effectively adopt the historical data of early degradation to learn the characteristics of the performance degradation trend and estimate the unknown further degraded performance states.

The proposed CLTD-CNN is an encoder-decoder structure that does not require a large amount of relevant domain expert knowledge and engineering experience so as to avoid errors caused by manual intervention. Specifically, the encoder part consists of a time-distributed 1D-CNN module and a 1D-ConvLSTM. The decoder part consists of a 1D-ConvLSTM and a simple time-CNN with residual connections. In order to better learn the characteristics of the performance degradation trend, the proposed structure introduces an auxiliary training part which allows the encoder part to efficiently encode the input data according to the performance degradation trend during the training process. Two sets of experiments on both the lateral damper and the yaw damper are carried out, and the experimental results demonstrate the validity and superiority of the proposed structure. Compared with other performance degradation estimation methods, CLTD-CNN obtains the minimum estimation error. The design ideas of CLTD-CNN presented in this paper also provide a reference for other areas of performance degradation estimation problems.

Future work focuses on the signal channel importance of performance degradation estimation. The study of signal channel importance enables the selection of critical channels for high-speed train signals to reduce computation and increase the speed of estimation without affecting the accuracy of estimation.

## Figures and Tables

**Table 1:** Summary of recent works on high-speed train bogie fault diagnosis and performance degradation estimation.

Content	Methods and references
Overview	[1]
	[2]
	[19]

Model-based methods	ALT method [9]
	DCLS calibration method [11]

Deep learning methods on fault diagnosis	Deep neural network [6]
	Residual-squeeze CNN [7]
	Multiscale CNN [8]
	CapsNet-based model [24]
	Bayesian deep learning [4]
	1D-CNN [5]
	Deep neural network [10]
	LSTM [25]
	1D-CNN [26]

Deep learning methods on performance degradation estimation	SDS-CNN [13]
	M-CRNN [14]

**Table 2:** Summary of recent works on deep learning.

Domain	References
Deep learning on image and video	[16, 17, 20, 27, 28]
Deep learning on signal	[21–23, 29–33]

**Table 3:** Hyperparameters of 1D-CNN in a time-distributed 1D-CNN module.

Layers	Parameters
1D-convolution layer	Filters: 64; kernel size: 3; stride: 2; activate function: ReLu
1D-convolution layer	Filters: 128; kernel size: 3; stride: 1; activate function: ReLu
1D-convolution layer	Filters: 128; kernel size: 3; stride: 1; activate function: ReLu
MaxPooling layer	Pool size: 2; stride: 2
1D convolution layer (Res)	Filters: 128; kernel size: 3; stride: 2; activate function: ReLu
1D-convolution layer	Filters: 256; kernel size: 3; stride: 1; activate function: ReLu
1D-convolution layer	Filters: 256; kernel size: 3; stride: 1; activate function: ReLu
MaxPooling layer	Pool size: 2; stride: 2
1D convolution layer (Res)	Filters: 256; kernel size: 3; stride: 2; activate function: ReLu

**Table 4:** Hyperparameters of 1D-ConvLSTM in encoder part.

Layers	Parameters
1D-ConvLSTM	Filters: 512; kernel size: 3; stride: 2; activate function: ReLu; return sequences: true

**Table 5:** Hyperparameters of 1D-CNN in auxiliary training part.

Layers	Parameters
1D-convolution layer	Filters: 512; kernel size: 3; stride: 1; activate function: ReLu
MaxPooling layer	Pool size: 2; stride: 2
1D-convolution layer	Filters: 1024; kernel size: 3; stride: 1; activate function: ReLu
MaxPooling layer	Pool size: 2; stride: 2
GlobalAveragePooling layer	—
Fully connected layer	Filters: 1024; dropout rate: 0.5
Fully connected layer	Filters: 1; dropout rate: 0.5

**Table 6:** Hyperparameters of decoder part.

Layers	Parameters
1D-ConvLSTM	Filters: 1024; kernel size: 3; stride: 2; activate function: ReLu; Return sequences: False
1D-convolution layer	Filters: 1024; kernel size: 3; stride: 1; activate function: ReLu
1D-convolution layer	Filters: 1024; kernel size: 3; stride: 2; activate function: ReLu
1D-convolution layer (Res)	Filters: 1024; kernel size: 3; stride: 2; activate function: ReLu
1D-convolution layer	Filters: 1536; kernel size: 3; stride: 2; activate function: ReLu
1D-convolution layer	Filters: 2048; kernel size: 3; stride: 2; activate function: ReLu
GlobalAveragePooling layer	—
Fully connected layer	Filters: 1024; dropout rate: 0.5
Fully connected layer	Filters: 1; dropout rate: 0.5

**Table 7:** Details of high-speed train signal channels.

Index	Description
1	lat.acc of the vehicle front part
2	lat.acc of the vehicle middle part
3	lat.acc of the vehicle rear part
4	ver.acc of the vehicle middle part
5	ver.acc of the vehicle front part
6	ver.acc of the vehicle rear part
7	lat.acc of the bogie 1 in pos. 1
8	ver.acc of the bogie 1 in pos. 1
9	lat.acc of the bogie 1 in pos. 4
10	ver.acc of the bogie 1 in pos. 4
11	lat.acc of the bogie 1 in the middle
12	ver.acc of the bogie 1 in the middle
13	lat.acc of the bogie 2 in pos. 5
14	ver.acc of the bogie 2 in pos. 5
15	lat.acc of the bogie 2 in pos. 8
16	ver.acc of the bogie 2 in pos. 8
17	lat.acc of the bogie 2 in the middle
18	ver.acc of the bogie 2 in the middle
19	lon.acc of the axle box 1
20	lat.acc of the axle box 1
21	ver.acc of the axle box 1
22	lon.acc of the axle box 2
23	lat.acc of the axle box 2
24	ver.acc of the axle box 2
25	lon.acc of the axle box 3
26	lat.acc of the axle box 3
27	ver.acc of the axle box 3
28	lon.acc of the axle box 4
29	lat.acc of the axle box 4
30	ver.acc of the axle box 4
31	lat.dis of the vehicle front part
32	ver.dis of the vehicle front part
33	lat.dis of the vehicle middle part
34	ver.dis of the vehicle middle part
35	lat.dis of the vehicle rear part
36	ver.dis of the vehicle rear part
37	lat.dis of the bogie 1 in pos. 1
38	ver.dis of the bogie 1 in pos. 1
39	lat.dis of the bogie 1 in pos. 4
40	ver.dis of the bogie 1 in pos. 4
41	lat.dis of the bogie 1 in the middle
42	ver.dis of the bogie 1 in the middle
43	lat.dis of the bogie 2 in pos. 5
44	ver.dis of the bogie 2 in pos. 5
45	lat.dis of the bogie 2 in pos. 8
46	ver.dis of the bogie 2 in pos. 8
47	lat.dis of the bogie 2 in the middle
48	ver.dis of the bogie 2 in the middle
49	lat.dis of the wheel-set 1
50	lat.dis of the wheel-set 2
51	lat.dis of the wheel-set 3
52	lat.dis of the wheel-set 4
53	Relative dis. of primary suspension in pos. 1
54	Relative dis. of primary suspension in pos. 8
55	Relative dis. of secondary suspension in pos. 1
56	Relative dis. of secondary suspension in pos. 8
57	Relative dis. of yaw damper in pos. 1
58	Relative dis. of yaw damper in pos. 8

Note: lat. = lateral, ver. = vertical, lon. = longitudinal, acc. = acceleration, dis. = displacement, and pos. = position.

**Table 8:** Details of the lateral damper data.

Training set				Test set		
Performance state (%)	Label (*Y*)	Label (*y*)	Number	Performance state (%)	Label (*y*)	Number
100, 95, 90, 85	[100, 95, 90, 85]	85	20000	95, 90, 85, 80	80	2000
95, 90, 85, 80	[95, 90, 85, 80]	80	20000	90, 85, 80, 75	75	2000
90, 85, 80, 75	[90, 85, 80, 75]	75	20000	85, 80, 75, 70	70	2000
85, 80, 75, 70	[85, 80, 75, 70]	70	20000	80, 75, 70, 65	65	2000
80, 75, 70, 65	[80, 75, 70, 65]	65	20000	75, 70, 65, 60	60	2000
75, 70, 65, 60	[75, 70, 65, 60]	60	20000	70, 65, 60, 55	55	2000
70, 65, 60, 55	[70, 65, 60, 55]	55	20000	65, 60, 55, 50	50	2000
65, 60, 55, 50	[65, 60, 55, 50]	50	20000	60, 55, 50, 45	45	2000
60, 55, 50, 45	[60, 55, 50, 45]	45	20000	55, 50, 45, 40	40	2000

**Table 9:** Details of the yaw damper data.

Training set				Test set		
Performance state (%)	Label (*Y*)	Label (*y*)	Number	Performance state (%)	Label (*y*)	Number
100, 95, 90, 85	[100, 95, 90, 85]	85	20000	95, 90, 85, 80	80	2000
95, 90, 85, 80	[95, 90, 85, 80]	80	20000	90, 85, 80, 75	75	2000
90, 85, 80, 75	[90, 85, 80, 75]	75	20000	85, 80, 75, 70	70	2000
85, 80, 75, 70	[85, 80, 75, 70]	70	20000	80, 75, 70, 65	65	2000
80, 75, 70, 65	[80, 75, 70, 65]	65	20000	75, 70, 65, 60	60	2000
75, 70, 65, 60	[75, 70, 65, 60]	60	20000	70, 65, 60, 55	55	2000
70, 65, 60, 55	[70, 65, 60, 55]	55	20000	65, 60, 55, 50	50	2000
65, 60, 55, 50	[65, 60, 55, 50]	50	20000	60, 55, 50, 45	45	2000
60, 55, 50, 45	[60, 55, 50, 45]	45	20000	55, 50, 45, 40	40	2000

**Table 10:** Results of experiments on step length *n*.

Step length *n*	Lateral damper		Yaw damper	
	MAE	RMSE	MAE	RMSE
2	3.96	4.35	4.37	4.88
3	1.98	2.50	2.17	2.68
4	**0.86**	**1.07**	**1.21**	1.46
5	0.93	1.14	1.22	**1.45**
6	0.89	1.17	1.25	1.60
7	1.19	1.43	1.38	1.72

The bold value means the minimum error of each case (column).

**Table 11:** Results of experiments on time-distributed 1D-CNN module.

Different cases	Lateral damper		Yaw damper	
	MAE	RMSE	MAE	RMSE
Without time-distributed 1D-CNN module	4.12	5.87	4.40	5.24
With time-distributed 1D-CNN module	**0.86**	**1.07**	**1.21**	**1.46**

The bold value means the minimum error of each case (column).

**Table 12:** Results of experiments on different RNN structures.

CLTD-CNN with different RNN structures	Lateral damper		Yaw damper	
	MAE	RMSE	MAE	RMSE
CLTD-CNN (with RNN)	3.56	4.01	5.02	5.79
CLTD-CNN (with LSTM)	3.24	3.51	3.15	3.47
CLTD-CNN (with GRU)	3.31	3.79	3.21	3.39
CLTD-CNN (with 1D-ConvGRU)	1.21	1.37	1.26	1.68
CLTD-CNN (with 1D-ConvLSTM)	**0.86**	**1.07**	**1.21**	**1.46**

The bold value means the minimum error of each case (column).

**Table 13:** Results of experiments on *λ*.

*λ*	Lateral damper		Yaw damper	
	MAE	RMSE	MAE	RMSE
0.0	2.16	2.31	3.16	3.32
0.1	1.04	1.16	1.39	1.77
0.2	**0.86**	**1.07**	1.21	**1.46**
0.3	0.94	1.12	**1.17**	1.50
0.4	1.18	1.35	1.48	1.76
0.5	1.83	2.15	1.91	2.35

The bold value means the minimum error of each case (column).

**Table 14:** Results of comparison experiments.

Method	Lateral damper		Yaw damper		GFLOPs	Average training	Inference time (s)
	MAE	RMSE	MAE	RMSE		Time per epoch (s)	(18000 test samples)
TCNN [39]	10.25	13.48	12.54	14.31	**0.074**	**15.2**	**5.7**
LSTM-AON [30]	8.01	10.40	13.67	15.39	1.044	180.7	92.5
BiGRU [33]	6.34	7.74	10.95	12.88	0.191	29.1	11.1
MDDNN [40]	3.87	4.46	4.33	5.17	0.518	88.9	40.4
SAE-LSTM [41]	2.44	2.96	3.35	4.04	0.807	151.4	72.1
M-CRNN [14]	2.41	3.52	2.74	3.34	0.380	74.3	33.4
SDS-CNN [13]	2.19	3.26	2.81	3.53	0.292	42.7	19.6
Proposed CLTD-CNN	**0.86**	**1.07**	**1.21**	**1.46**	0.322	57.1	26.2

The bold value means the minimum error of each case (column).

## Data Availability

The experimental data used to support the findings of this study are available from the corresponding author upon request.
